# Improved method for stress detection using bio-sensor technology and machine learning algorithms

**DOI:** 10.1016/j.mex.2024.102581

**Published:** 2024-01-23

**Authors:** Mohd Nazeer, Shailaja Salagrama, Pardeep Kumar, Kanhaiya Sharma, Deepak Parashar, Mohammed Qayyum, Gouri Patil

**Affiliations:** a Vidya Jyothi Institute of Technology, Hyderabad 500075, India; b Computer Information System, University of the Cumberland's, Williamsburg, KY 40769, USA; c Anurag Univerisity, Venkatapur, Ghakesar Rd, Hyderabad, Telengana 500088, India; d Symbiosis Institute of Technology Pune, Symbiosis International (Deemed) University, Pune 411021, India; e King Khaled University, Abha 61421, Saudi Arabia; f Muffakhamjah College of Engineering and Technology, Hyderabad 500034, India

**Keywords:** Bio-sensor, Stress, ECG, GSR, Machine learning, Machine learning algorithms

## Abstract

Maintaining an optimal stress level is vital in our lives, yet many individuals struggle to identify the sources of their stress. As emotional stability and mental awareness become increasingly important, wearable medical technology has gained popularity in recent years. This technology enables real-time monitoring, providing medical professionals with crucial physiological data to enhance patient care. Current stress-detection methods, such as ECG, BVP, and body movement analysis, are limited by their rigidity and susceptibility to noise interference. To overcome these limitations, we introduce STRESS-CARE, a versatile stress detection sensor employing a hybrid approach. This innovative system utilizes a sweat sensor, cutting-edge context identification methods, and machine learning algorithms. STRESS-CARE processes sensor data and models environmental fluctuations using an XG Boost classifier. By combining these advanced techniques, we aim to revolutionize stress detection, offering a more adaptive and robust solution for improved stress management and overall well-being.

• In the proposed method, we introduce a state-of-the-art stress detection device with Galvanic Skin Response (GSR) sweat sensors, outperforming traditional Electrocardiogram (ECG) methods while remaining non-invasive
• Integrating machine learning, particularly XG-Boost algorithms, enhances detection accuracy and reliability.
• This study sheds light on noise context comprehension for various wearable devices, offering crucial guidance for optimizing stress detection in multiple contexts and applications.

## Graphical abstract

**Figure:**
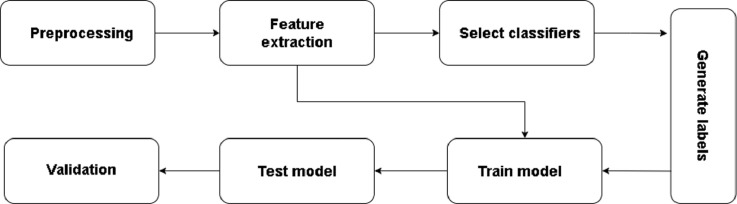

Specification table

**Table:** 

Subject area:	Engineering
More specific subject area:	Detection, Biosensor
Name of your method:	Machine learning algorithms
Name and reference of original method:	N. Rashid, T. Mortlock and M. A. A. Faruque, “Stress Detection Using Context- Aware Sensor Fusion From Wearable Devices,” in *IEEE Internet of Things Journal*, vol. 10, no. 16, pp. 14,114–14,127, 15 Aug.15, 2023, doi: 10.1109/JIOT.2023.3265768
Resource availability:	https://ieeexplore.ieee.org/document/10,097,874

## Method details

**Fig. 1:**
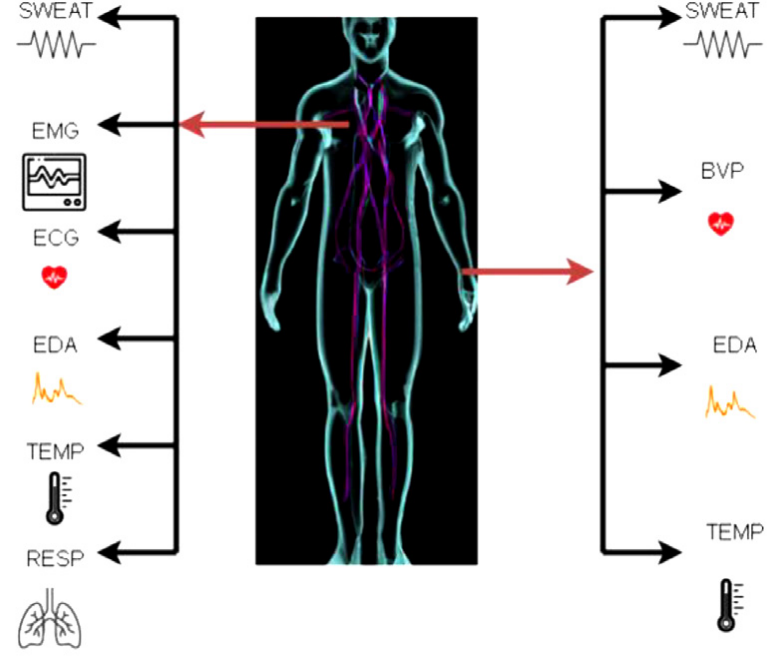
Deployment of the various wrist and chest sensors.

**Fig. 2:**
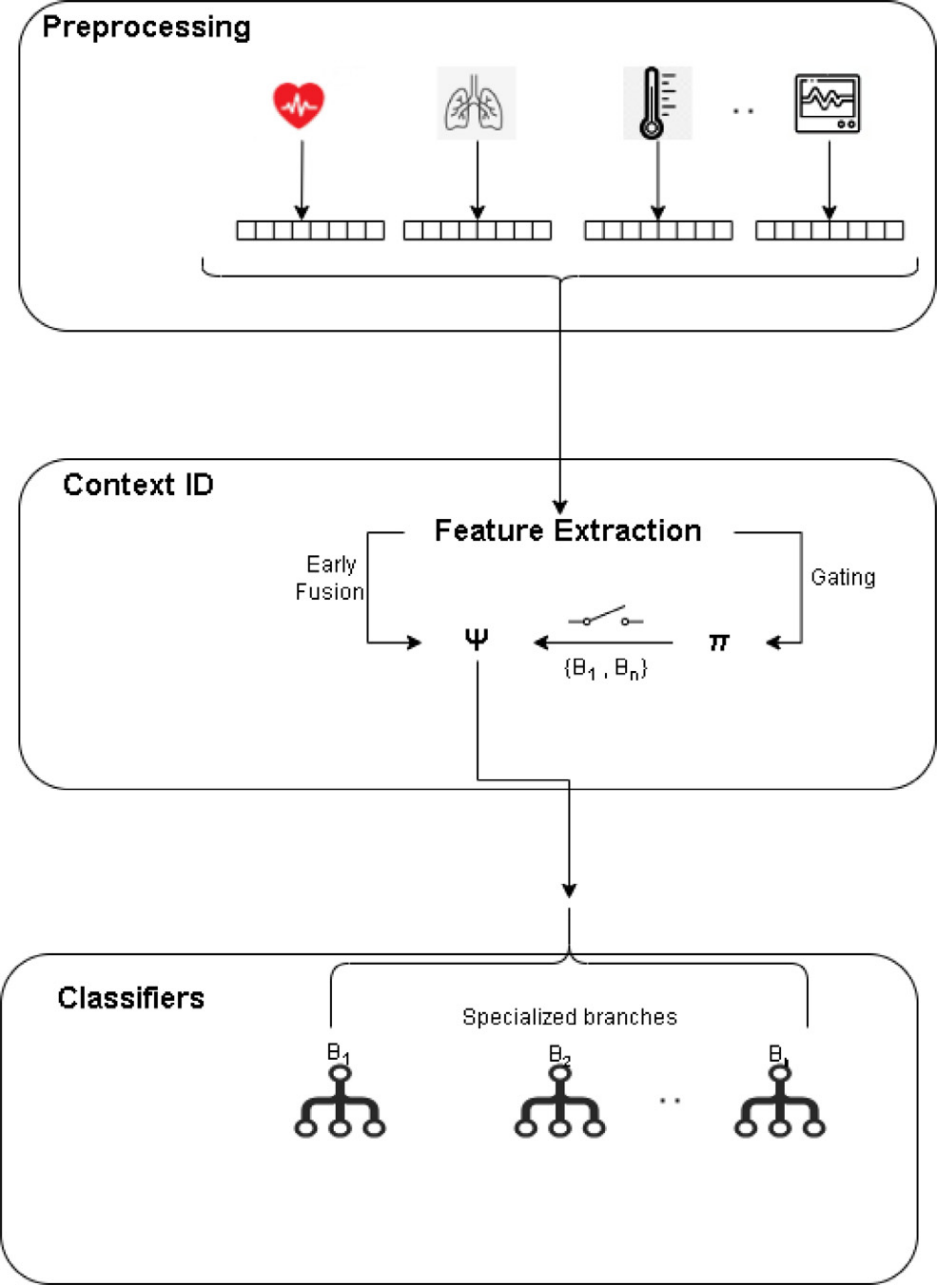
Stress-care architecture.

In our fast-paced lives, optimal stress management is crucial. Wearable medical technology has surged, but existing stress-detection methods face limitations [1]. Introducing STRESS-CARE, a versatile, hybrid sensor revolutionizing stress detection for enhanced well-being [2]. The proliferation of wearable medical devices has been driven by technological advancements and the rise of the Internet of Things (IoT). These devices enable continuous remote monitoring of vital physiological signals, revolutionizing healthcare [3], [4], [5], [6]. Wearable health technology, particularly in stress detection, is burgeoning, with a significant market projection by 2025 [7], [8], [9], [10], [11], [12]. Researchers utilize various machine learning techniques, such as Convolutional Neural Networks (CNN) and Support Vector Machines (SVM), to tackle the challenge of linking stress levels with sensor data. Integrating multiple sensor types and addressing sensor noise remains a challenge. Our study introduces a cutting-edge stress detection device using Galvanic Skin Response (GSR) sweat sensors, enhancing accuracy through XG-Boost algorithms. We also introduce a context-aware sensor approach, evaluating existing research and datasets. The WSADS dataset, encompassing amusement and stress states, stands out. Our findings align with the importance of context modelling and sensor fusion in wearable technology. Introducing our STRESS-CARE framework, depicted in Fig. 1. Whereas, Fig. 2 consists of three core elements: pre-processing, context identification, and branch classifiers. STRESS-CARE's primary objective is to categorize stress by analysing sensor data from specific time intervals. We employ a multi-branch structure, where each “branch” represents a stress detection classifier utilizing diverse sensor configurations. The pivotal role of context identification lies in determining the branches to activate. In cases where multiple units are selected, we employ late fusion to amalgamate their stress predictions [13], [14], [15], [16], [17].

STRESS-CARE is engineered to accommodate data inputs from a variety of physiological sensors, encompassing both heterogeneous and homogeneous types. Typically, raw, unprocessed data necessitates pre-processing to ready them for analysis. Context identification is instrumental in predicting the most suitable branch classifier, taking into account input sensor characteristics that aptly represent the system's context. Context modelling is indispensable in comprehending how different sensors respond under varying conditions and in optimizing the placement of wearable devices on the body. For wrist-worn devices, we leverage motion as a vital context indicator, initially extracting Accelerometer (ACC) characteristics. In contrast, for chest-worn wearables, our focus shifts to modeling muscle contraction context, detected via the Electromyogram (EMG) signal, alongside stress levels. Consequently, we extract EMG features for context modeling in chest-worn devices. The gating model then processes these features to determine which branches to activate. Feature extraction for other sensor modalities occurs subsequently, ensuring that the most relevant sensors are engaged based on context. This adaptive approach enhances stress detection while streamlining computational load and complexity. The Test Model trains a classifier that selects an appropriate branch classifier based on whether the device is worn on the wrist or chest, utilizing ACC/EMG features as input. For wrist-worn devices, the RF classifier is employed for both 2-class and 3-class classification, narrowing down the options to three branches. WB1= {EDA, BVP, TEMP}, WB2 = {sweat, EDA, BVP}, and WB3= {EDA, BVP}. We employ XG Boost classifiers to select three branches for 2-class classification for chest-worn devices. We opt for a Decision Tree (DT) classifier for the proposed test model due to its compactness and minimal impact on our design. Performance-Computation Trade-off: STRESS-CARE's ability to balance performance and computational constraints is integral.

**Fig. 3:**
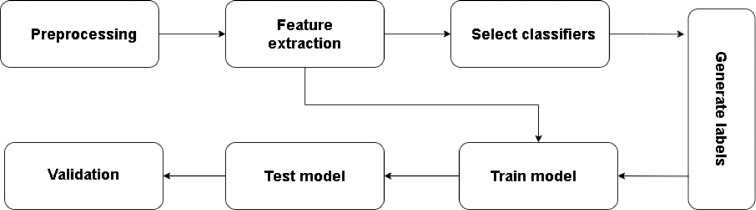
Stress care training and implementation procedure.

The test model outputs prediction probabilities for the available branches, with ‘b’ representing the branch with the highest probability. ‘b’ falls within the range of 1 to 0, signifying the threshold at which non-maximum branches are selected. Higher values permit the selection of more branches, while lower values indicate stricter computational limitations. For instance, a value of 0 implies the selection of the highest probability branch from the gating classifier, whereas a value of 1 implies selecting all branches. Branch Classifiers: The segment is subsequently classified using the relevant branch classifier(s). For both 2-class and 3-class classification in our approach, we employ Decision Tree (DT), Random Forest (RF), Support Vector Machine (SVM), and XGBoost classifiers for all three branches of wrist modalities. We utilize Decision Tree (DT), Random Forest (RF), and XGBoost classifiers for the two-class categorization of chest modalities. Now only uses chest sensors or wrist sensors to function, but our solution can integrate both sets of branches with slight changes to the context identification module. The late fusion method requires a classification prediction from each chosen branch as an input. Stress care Training and Implementation Procedure is shown in Fig. 3.

### Dataset

The experimental setup of STRESSCARE on a stress detection dataset using wearable health devices are presented in this part. We begin by outlining the evaluation dataset. Secondly, it describes the development and application of our models. Thirdly, we discuss our evaluation measures and review the findings of our experiments. The publicly accessible WESAD dataset is used to validate STRESS-CARE [17]. This dataset was chosen because it incorporates information about the Sweat, ECG, GSR for wearables that may be worn on the wrist and the chest, which makes it the perfect dataset for comprehending the different context devices worn on various body parts. There is data for a total of 16 subjects in the dataset from sensors worn on the wrist (Empatica E4) and the chest (RespiBAN). RespiBAN uses the SWEAT, RESP, ECG, EMG, TEMP and EDA chest sensors. The Empatica E4′s wrist sensors are SWEAT, BVP, EDA, and TEMP. Three different classifications relating to emotional states are present in the dataset: baseline (neutral), amusement, and stress. Baseline and amusement are combined in the non-stress class of the two-class dilemma [18], [19], [20], [21], [22].

Wrist devices: considers the stress, GSR, heart rate during rest (38 BPM) or high heart rates brought on by exercise or tachycardia (220 BPM). Chest devices: The other signals (EDA, ECG, RESP, EMG, GSR, SWEAT and TEMP) are subsequently smoothed using an order of 3 and a window size of 10. The ECG signal is further filtered, similarly to wrist BVP, by a band-pass Butterworth filter. A window of 50 s of data with a sliding duration of 8 s after it segments the filtered signals from the chest and wrist [23], [24], [25], [26]. In total, 6657 segments are generated for each signal across all WESAD dataset individuals as an outcome of this approach. The same chest and wrist sensor features that were used in [17] are extracted here, including absolute integrals, peak and power frequencies, slope and dynamic ranges, correlations, slope, standard deviations and mean/standard deviations. For wrist modalities combinations are in Tables 1 and 2. For chest modalities, we tried Thirty various combinations of chest sensors as input branches as shown in Table 3. We train utilising various combinations of input sensor data for each branch classifier within STRESS-CARE. We use three alternative early fusion wrist sensor combinations as input branches for Wrist Modalities: We test each branch using one of four machine learning classifiers: Decision Tree (DT), Random Forest (RF), XG Boost (XG), SVM. WB1=BVP, TEMP, EDA; WB2=SWEAT, EDA, BVP; and WB3= EDA, BVP.

**Table 1:** Performance (in%) of wrist devices for 3-class (Baseline VS Stress VS Amusement).

Modality Used	Decision Tree		Random Forest		XGBoost		SVM	
	F1 Score	Accuracy	F1 Score	Accuracy	F1 Score	Accuracy	F1 Score	Accuracy
WB1={BVP,EDA,TEMP}	54.18	60.3	73.82	77.51	79.71	89.92	66.74	76.24
WB2={Sweat, BVP,EDA}	60.12	50.38	78.31	79.62	79.1	88.24	67.74	79.82
WB3={BVP,EDA}	55.86	59.68	75.1	74.92	76.24	84.56	65.12	77.86

**Table 2:** Performance (in%) of wrist devices for 2-class (Stress VS Non Stress).

Modality Used	Decision Tree		Random Forest		XGBoost		SVM	
	F1 Score	Accuracy	F1 Score	Accuracy	F1 Score	Accuracy	F1 Score	Accuracy
WB1={BVP,EDA,TEMP}	74	83.4	85.1	89.9	92.16	94.53	84.25	88.92
WB2={Sweat,BVP,EDA}	69.98	77.8	86.28	90.2	93.25	96.12	84.71	93.25
WB3={BVP,EDA}	80.94	85.6	86.42	89.76	90.1	93.65	90.08	91.42

**Table 3:** Performance (in %) of Chest devices for binary class (Stress VS Non Stress).

Modality Used	Decision Tree		Random Forest		XGBoost	
	F1 Score	Accuracy	F1 Score	Accuracy	F1 Score	Accuracy
CB1={ECG,RESP,EMG,EDA,TEMP}	71.1	73.8	80.04	84.09	83.6	88.59
CB2={Sweat,ECG,RESP,EMG,TEMP}	64.2	70.11	80.1	83.1	85.06	89.1
CB3={Sweat,ECG,RESP,EMG}	65.82	73.6	76.15	78.15	78.1	82.1
CB4={Sweat,ECG,RESP,EMG}	63.9	70.1	72.58	77.6	75.6	80.09
CB5={Sweat,ECG,RESP,EDA}	69.39	79.8	85.4	88.5	88.42	92.4
CB6={Sweat,ECG,EMG,TEMP}	64	72.22	82.13	83.12	85.15	90.1
CB7={Sweat,ECG,EMG,EDA}	70.42	75.72	85.5	88.62	88.1	93.05
CB8={Sweat,RESP,EDA,TEMP}	66.7	72.26	83.03	85.09	85.1	92.02
CB9={Sweat,ECG,EDA,TEMP}	63.1	69.75	85.47	88.5	88.4	93.5
CB10={ECG,RESP,EMG,TEMP}	50.51	52.7	78.88	80.6	81.1	86.09
CB11={ECG,RESP,EMG,TEMP}	70.81	75.5	83.24	85.3	85.2	89.22
CB12={ECG,EMG,EDA,TEMP}	70.3	73.25	85.05	87.1	90.1	93.05
CB13={ECG,RESP,EDA,TEMP}	71.6	73.5	79.44	80.5	83.4	90.5
CB14={RESP,EMG,EDA,TEMP}	69.8	71.11	85.82	88.8	89.1	92.6
CB15={Sweat,EDA,TEMP}	68.84	71	83.5	85.52	88.64	91.62
CB16={Sweat,EMG,EDA}	70.7	73.11	79.95	81.97	85.9	90.02
CB17={Sweat,RESP,EDA}	68.5	75.4	79.2	81.22	83.06	88.52
CB18={Sweat,ECG,RESP}	64.1	70.19	75.3	78.1	82.1	88.09
CB19={Sweat,RESP,EMG}	66.78	72.5	69.44	73.5	73.5	79.62
CB20={Sweat,ECG,EDA}	70.5	75.85	87.11	89.1	90.55	95.1
CB21={ECG,RESP,EMG}	63.4	74.81	69.82	72.8	72.9	98.88
CB22={ECG,EDA,TEMP}	75.1	66.28	80.4	85.2	88.5	93.1
CB23={ECG,RESP,EDA}	73.18	76.06	85.69	89.7	89.72	92.7
CB24={ECG,EMG,EDA}	78.8	74.45	75.27	78.3	80.23	84.22
CB25={RESP,EMG,EDA}	59.6	75.26	79.59	82.77	83.6	86.52
CB26={RESP,EDA,TEMP}	66.55	63.4	82.65	85.7	88.63	92.6
CB27={EMG,EDA,TEMP}	69.9	70.23	70.92	78.9	75.93	80.9
CB28={Sweat,RESP}	74.95	75.2	68.4	70.42	74.6	80.72

### Performance evaluation parameters

To guarantee a fair comparison with the original WESAD study, we used these classifiers. As previously mentioned, the WESAD dataset has a very uneven distribution of segments by class. Since this is the reason, in order to evaluate the performance of classification, we use both the accuracy and F1 score. To make sure that comparisons with other works are fair.

(1) Accuracy=(TP+TN)/(TP+FP+TN=FN)

(2) $$\text{Macro}\;F_{1}=\frac{1}{n_{c}}\left\{\sum_{i}^{n_{c}}2*\;\frac{x_{i}.y_{i}}{x_{i}+\;y_{i}}\right\}$$

(3) $$X_{I}=\text{TP}/(\text{TP}+\text{FP})$$

(4) $$Y_{I}=\text{TP}/(\text{TP}+\text{FN})$$

Here TN= True Negative, TP = True Positive, FN = False Negative, FP = False Positive.

*X* = precision, *Y* = recall.

### Method validation

**Fig. 4:**
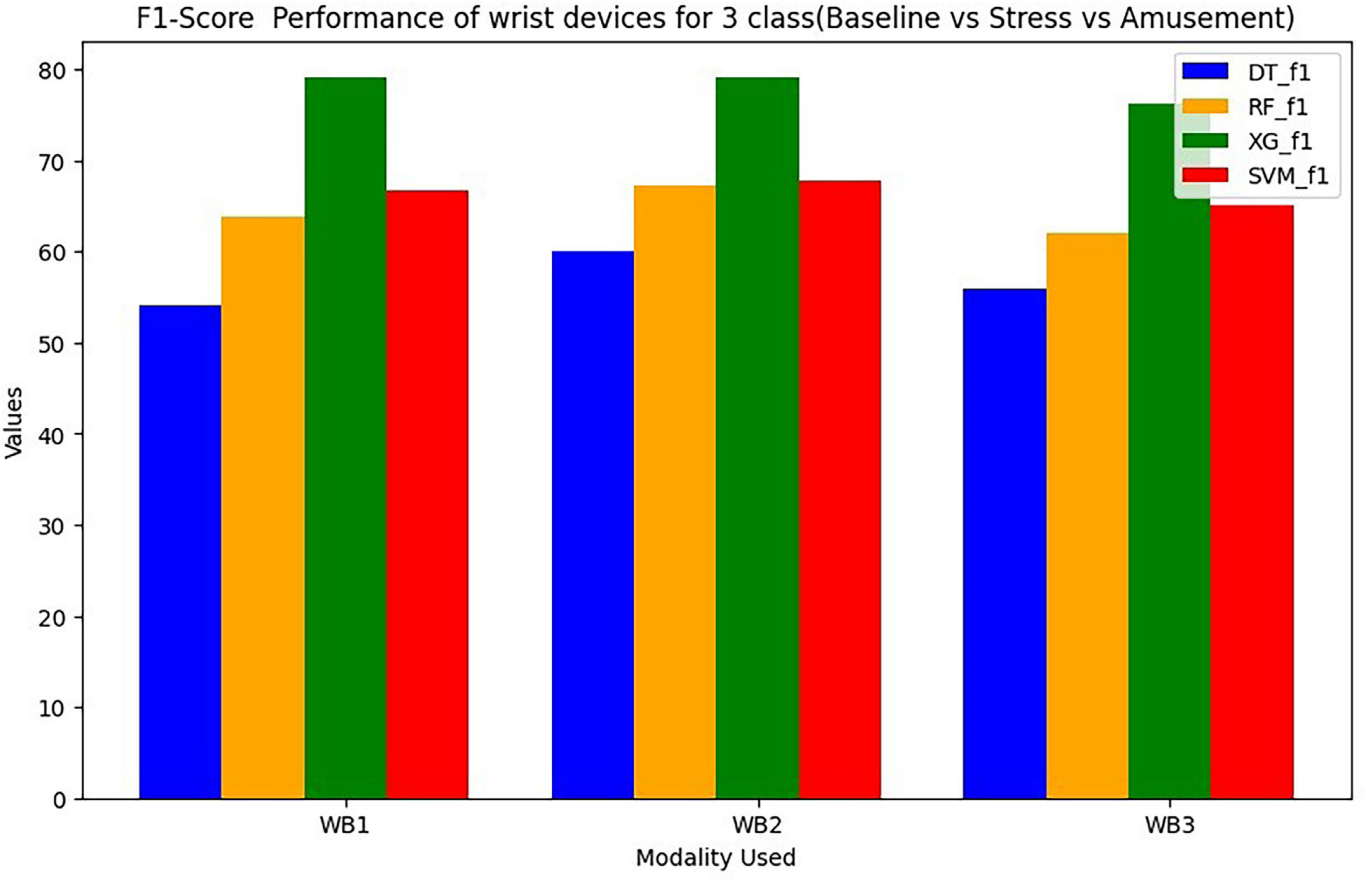
Comparing the overall F1 score and accuracy performance by using 3-class wrist data.

**Fig. 5:**
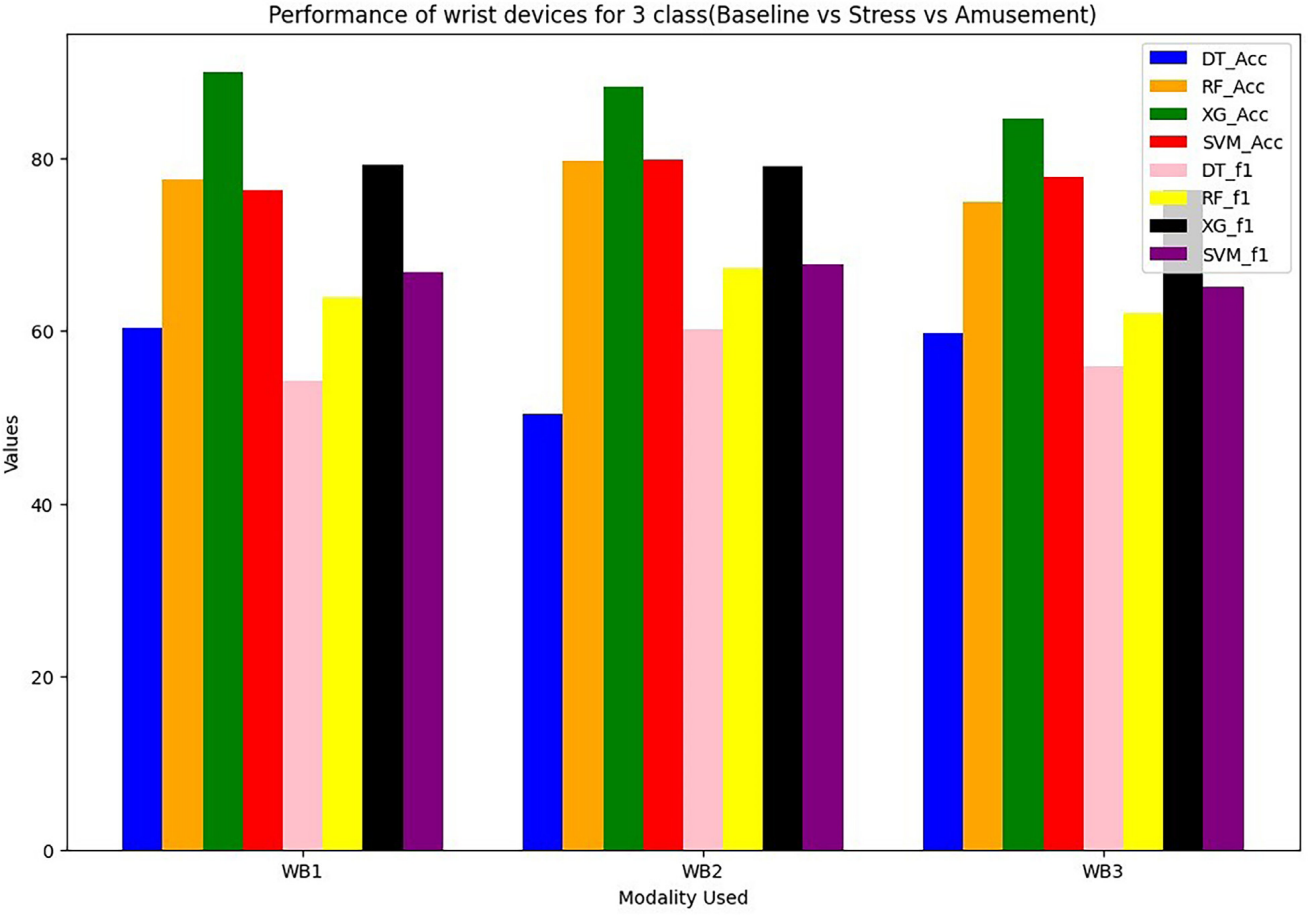
Comparing the overall performance of 3-class wrist data.

The performance evaluation of STRESS-CARE for detecting stress in 2-class and 3-class categorization utilising chest and wrist devices is presented in this part. For evaluation of wrist modalities performance, we conducted a performance analysis of various classifiers for both 2-class and 3-class issues using different input branches, where each branch represents distinct combinations of input sensors. Tables 1 and 2 present the finding of the proposed method. Among these, the XGBoost classifier for branches WB1, WB2, and WB3 consistently outperforms others, demonstrating superior or competitive performance for both 2-class and 3-class classification tasks. When trained with XGBoost, these branches achieved minimal training loss, leading us to select XGBoost as the classifier for these specific input branches. It achieves a macro F1 score of 90.1% and an accuracy of 93.65%, as demonstrated in Fig. 4. Furthermore, Fig. 5 illustrates that our STRESS-CARE technique surpasses other comparable methods [16], [17], [18], branch classifiers, and conventional late fusion techniques in 3-class classification. It achieves remarkable performance with macro F1 scores and accuracy reaching 79.71% and 89.72%, respectively. While STRESS-CARE outperforms [14] in accuracy, the two methods attain similar macro F1 scores. This parity in macro F1 scores is because leverages both chest and wrist sensors for stress classification. In the realm of 2-class classification, STRESS-CARE also outperforms related studies [15,16], branch classifiers, and conventional fusion methods.

For evaluation of chest modalities performance, we evaluated various classifiers for 2-class issues using different input branches, with each branch representing distinct combinations of input sensors (Table 3). Among the 2-class classifiers, the XGBoost classifier performs superior for branches CB1, CB12, CB14, and CB24, consistently outperforming others. This preference for XGBoost stems from its minimal training loss for these specific input branches during training and the proposed STRESS-CARE method outperforms other related works [16,17], branch classifiers, and conventional late fusion techniques in terms of accuracy and macro F1 scores. Importantly, this study highlights that STRESS-CARE maintains its superior performance even with motion-based context understanding. These plots emphasize that mobility is not always the optimal choice for contextual understanding in the context of stress detection using chest modality.

**Fig. 6:**
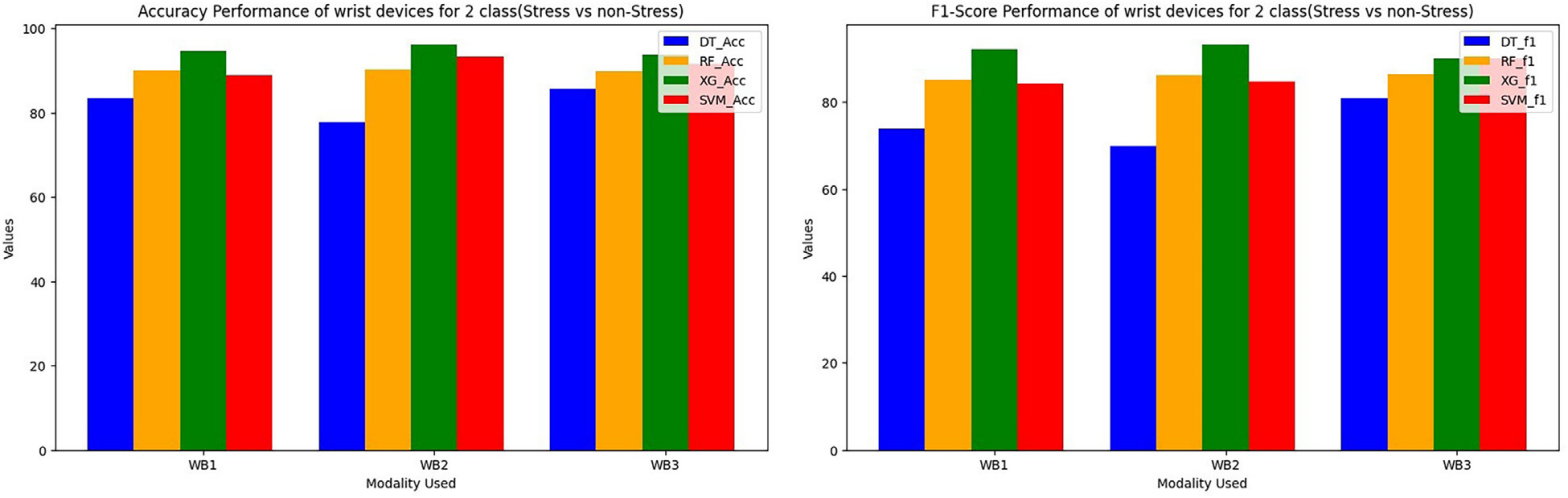
Comparing the overall F1 score and accuracy performance by using 2-Class wrist data.

**Fig. 7:**
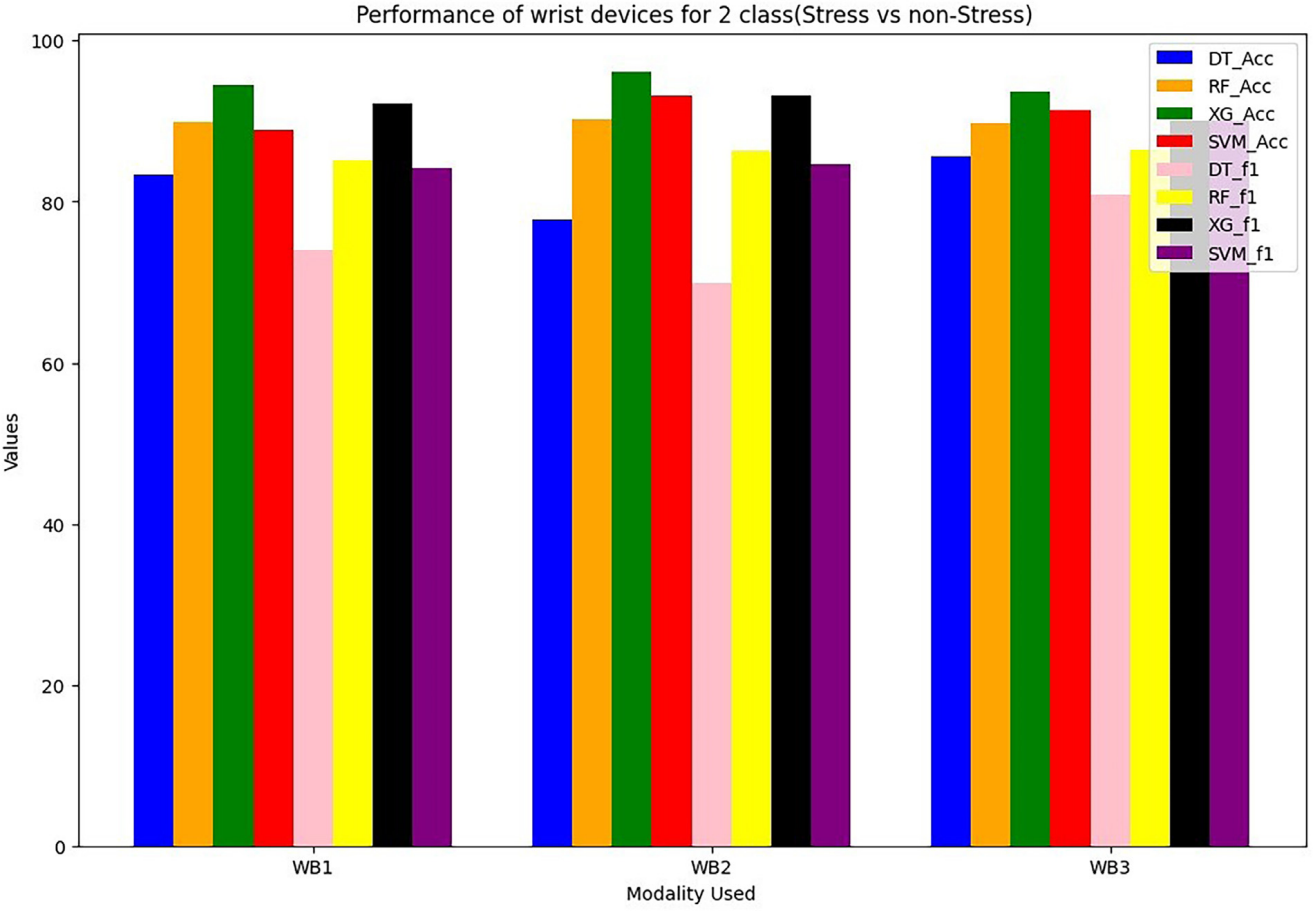
Comparing the overall performance of 3-Class wrist data.

**Fig. 8:**
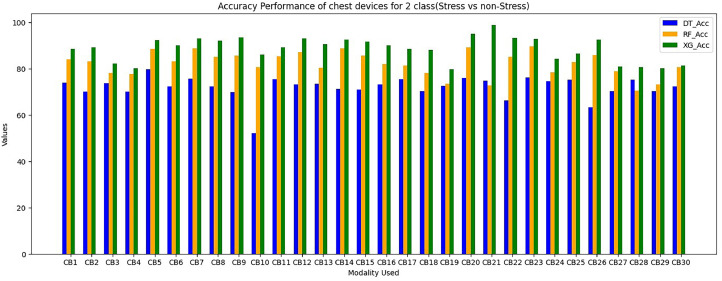
Comparing the Accuracy performance of 2-Class chest data.

**Fig. 9:**
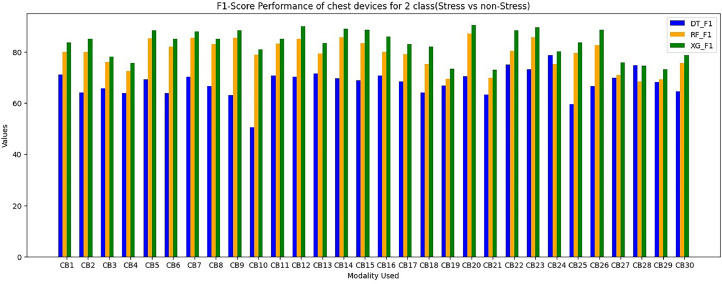
Comparing the F1 Score performance of 2-Class chest data.

**Fig. 10:**
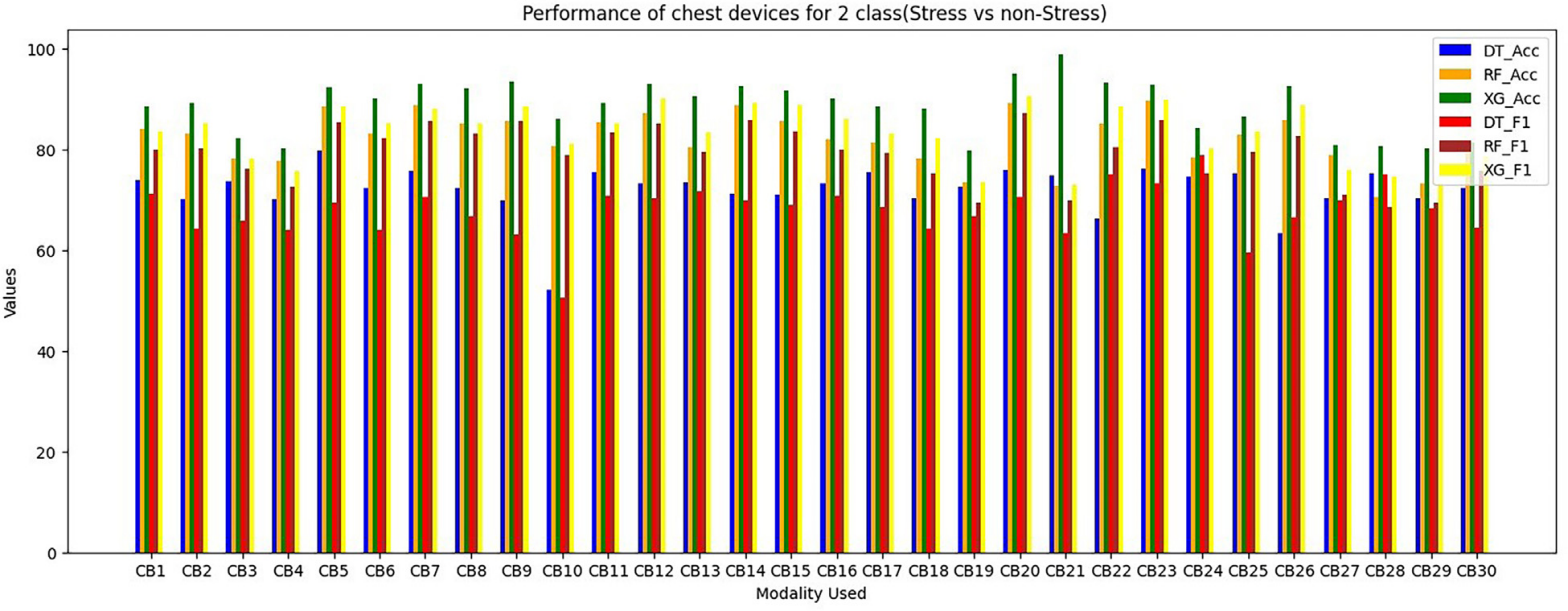
Comparing the overall performance of 2-Class chest data.

The proposed STRESSCARE model performs better than comparable works, branch classifiers, and other conventional late fusion algorithms in terms of both F1 score and accuracy. Fig. 6 comparing the overall F1 score and accuracy performance by using 2-Class wrist data. On the other side, Fig. 7 compares different wrist devices utilizing various algorithms, including decision trees, random forests, SVM, and our proposed XG Boost. Clearly, the proposed algorithm outperforms existing ones. Figs. 8 and 9 display the accuracy and F1 score comparisons of various chest devices using established algorithms such as decision trees, random forests, SVM, and our proposed XG Boost. Fig. 10 compares various chest devices employing different combinations. The performance (in%) of various chest devices for binary class using decision tree, random forest, and XGBoost is depicted in Table 3. Our XG Boost algorithm exhibits superior performance, as evidenced by its accuracy and F1 score surpassing that of current methods. This underscores its efficiency in accurately classifying diverse bio-sensor values. Notably, our algorithm excels across both wrist and chest devices, positioning it as a promising solution for improving stress detection and the analysis of bio-sensor data.

Our experimental findings introduce STRESS-CARE, a ground-breaking stress detection method combining sweat sensors and ECG devices. It dynamically adapts sensor fusion, revolutionizing wearable health monitoring. Results show wrist-mounted sensors benefit from motion context. STRESS-CARE excels with XG Boost, achieving top performance on the Wearable Stress and Affect Detection dataset. Wrist sensors achieve 89.92% (3-class) and 96.12% (2-class) accuracy, while chest sensors reach 98.88% (2-class). These findings highlight STRESS-CARE's potential to enhance stress detection accuracy and broaden its utility in wearable health monitoring. Future research will explore sensor location impact, energy efficiency, and extend its applications to diverse healthcare fields, improving global well-being. The stress detection system, STRESS-CARE, faces challenges such as susceptibility to environmental influences, requiring ongoing refinement of its hybrid approach. Ensuring user comfort and addressing potential sensor-related issues are additional considerations for real-world application.

## CRediT authorship contribution statement

**Mohd Nazeer:** Conceptualization, Methodology, Software, Writing – original draft. **Shailaja Salagrama:** Methodology, Funding acquisition. **Pardeep Kumar:** Methodology, Software. **Kanhaiya Sharma:** Conceptualization, Visualization, Funding acquisition. **Deepak Parashar:** Conceptualization, Visualization, Supervision. **Mohammed Qayyum:** Writing – review & editing. **Gouri Patil:** Writing – review & editing.

## Declaration of competing interest

The authors declare that they have no known competing financial interests or personal relationships that could have appeared to influence the work reported in this paper.

## Data Availability

Data is publicly available.
